# A pin-fasten grafting method provides a non-sterile and highly efficient method for grafting Arabidopsis at diverse developmental stages

**DOI:** 10.1186/s13007-015-0081-7

**Published:** 2015-07-08

**Authors:** Nien-Chen Huang, Tien-Shin Yu

**Affiliations:** Institute of Plant and Microbial Biology, Academia Sinica, Taipei, 11529 Taiwan

**Keywords:** Arabidopsis pin-fasten grafting, Epicotyls, Hypocotyls, 5(6)-Carboxyfluorescein diacetate (CFDA), Symplastic connection, Developmental stages

## Abstract

**Background:**

Higher plants have evolved sophisticated communication systems to integrate environmental stimuli into their developmental programs. Grafting provides a powerful technique to examine transportation and systemic effects of mobile molecules. In Arabidopsis, many grafting approaches have been developed to investigate systemic molecules. However, these methods are usually limited to specific developmental stages or require sterilized conditions. To broaden the application of grafting for examining systemic signals at diverse developmental stages, we developed an Arabidopsis pin-fasten grafting method with insect pins used to assemble stocks and scions.

**Results:**

We report the step-by-step protocol of Arabidopsis pin-fasten grafting. Arabidopsis wild-type or *gl1*-*1* plants were grown under long- or short-day conditions. Insect pins were inserted into *gl1*-*1* scions at different developmental stages for grafting onto epicotyls or hypocotyls of stocks. Successfully grafted scions with newly developed glabrous leaves were observed at 14 days after grafting. Further longitudinal sections of the graft union showed well-connected vascular tissues between grafted plants. Use of fluorescent phloem-limited dye carboxyfluorescein diacetate in grafted plants demonstrated a symplastic connection established at 6 days after grafting and almost fully developed at 8 days.

**Conclusions:**

Our method provides a simple and robust approach to grafting Arabidopsis at different developmental stages. Sterilized conditions are not required, which greatly improves the success of grafting and plant growth.

**Electronic supplementary material:**

The online version of this article (doi:10.1186/s13007-015-0081-7) contains supplementary material, which is available to authorized users.

## Background

Grafting is an ancient agricultural technique that has been widely used to improve agricultural traits without genetic modification for more than 1,000 years. Cultivars with pathogen resistance or stress tolerance are usually used as rootstocks to improve the growth vigor of cultivars with high economic value. Grafting has also been used to break the juvenile stage to promote flowering in woody species or proposed to resolve the limitation of genetically modified plants [1]. It has been observed that the growth behavior of scions is sometimes affected after grafting, which suggests that signals derived from the stocks may traffic long-distance to affect scion growth [1–3]. Thus, grafting also provides an effective tool to investigate long-distance signaling in the regulation of plant development.

In the model system Arabidopsis, the rosette-type and diminutive stature of plants may impede the manipulation of grafting. However, in the past decades, several grafting approaches, including inflorescence grafting [4–7], micrografting (with 3- to 9-day-old plants) [8, 9], seedling grafting (with 10- to 12-day-old plants) [10, 11], mature plant grafting [12], cotyledon grafting [13], and root grafting [14], have been developed to examine long-distance signals in Arabidopsis. The Arabidopsis inflorescence, which contains long internodes and sufficient diameter, provides ideal material for grafting. In inflorescence grafting, primary inflorescences with a wedge-style or flat surface cut are used as scions to graft onto inflorescences of rootstock. The graft junctions are tightly sealed with polyethylene tubes and parafilm to provide physical support [4–7]. Because of the ease of manipulation and high success rate, inflorescence grafting has become a reliable method to study long-distance trafficking in Arabidopsis. This approach has been used to study leaf-derived signals in inflorescence development [4] or long-distance movement of mRNA [6, 15]. However, the tissues used for inflorescence grafting are restricted to the reproductive stage, which limits its use for studying long-distance signaling regulating vegetative growth or the onset of flowering.

Micrografting techniques with young seedlings have been developed to investigate long-distance signals involved in shoot branching of Arabidopsis [8] or other systemic effects. In micrografting, 3- to 9-day-old Arabidopsis seedlings grown under sterilized conditions are used for grafting. Scions with a horizontal or wedge cut are assembled with hypocotyls of rootstocks to form transverse or wedge grafts [8, 9]. More complicated two-shoot grafting is achieved by inserting the wedge-cut scions into slits made on hypocotyls of rootstocks to form Y-shaped grafts [8]. These techniques have been used to identify many mobile signals, including florigen [16–18], mobile miRNA or siRNA [19–22], and long-distance signals in shoot branching [8, 23]. Other Arabidopsis grafting techniques used with specialized tissues such as roots or cotyledons have been developed [13, 14].

Many systemic informative molecules travel through phloem. The manipulation of phloem translocation may greatly alter the transportation of mobile molecules in phloem and thereby influence their detection and their effects. Phloem transport is highly dynamic. The direction of phloem translocation is governed by sink-source strength and phyllotaxy and can be manipulated by defoliation [24–26]. As the sink leaves of scions turn into source leaves, they start to transport nutrient and signaling molecules into phloem. Therefore, the scion tissues gradually receive reduced phloem translocation derived from stocks [6]. In addition, the phloem translocation in lower leaves mainly transports to root and in upper leaves mainly to the apical meristem [27]. The position of grafted tissues and how stocks and scions are connected may affect the direction of phloem translocation. Therefore, different grafting approaches may result in contradictory detection of mobile signals [11, 17, 18].

In this paper, we describe a protocol for Arabidopsis pin-fasten grafting that is modified from previous Arabidopsis seedling grafting [10, 11]. In pin-fasten grafting method, soil-grown scions were pin-inserted to graft onto epicotyls or hypocotyls of stocks. This method can be used under long- or short-day growth conditions or various developmental stages. The vascular connection between scions and stocks was confirmed by longitudinal sectioning and microscopy. We verified the symplastic connection between scions and stocks by using phloem-limited dye. Our approach provides a sterile-free and highly efficient method for grafting under a wide range of developmental stages.

## Results and discussion

### Arabidopsis pin-fasten grafting under long-day (LD) growth conditions

**Figure 1:**
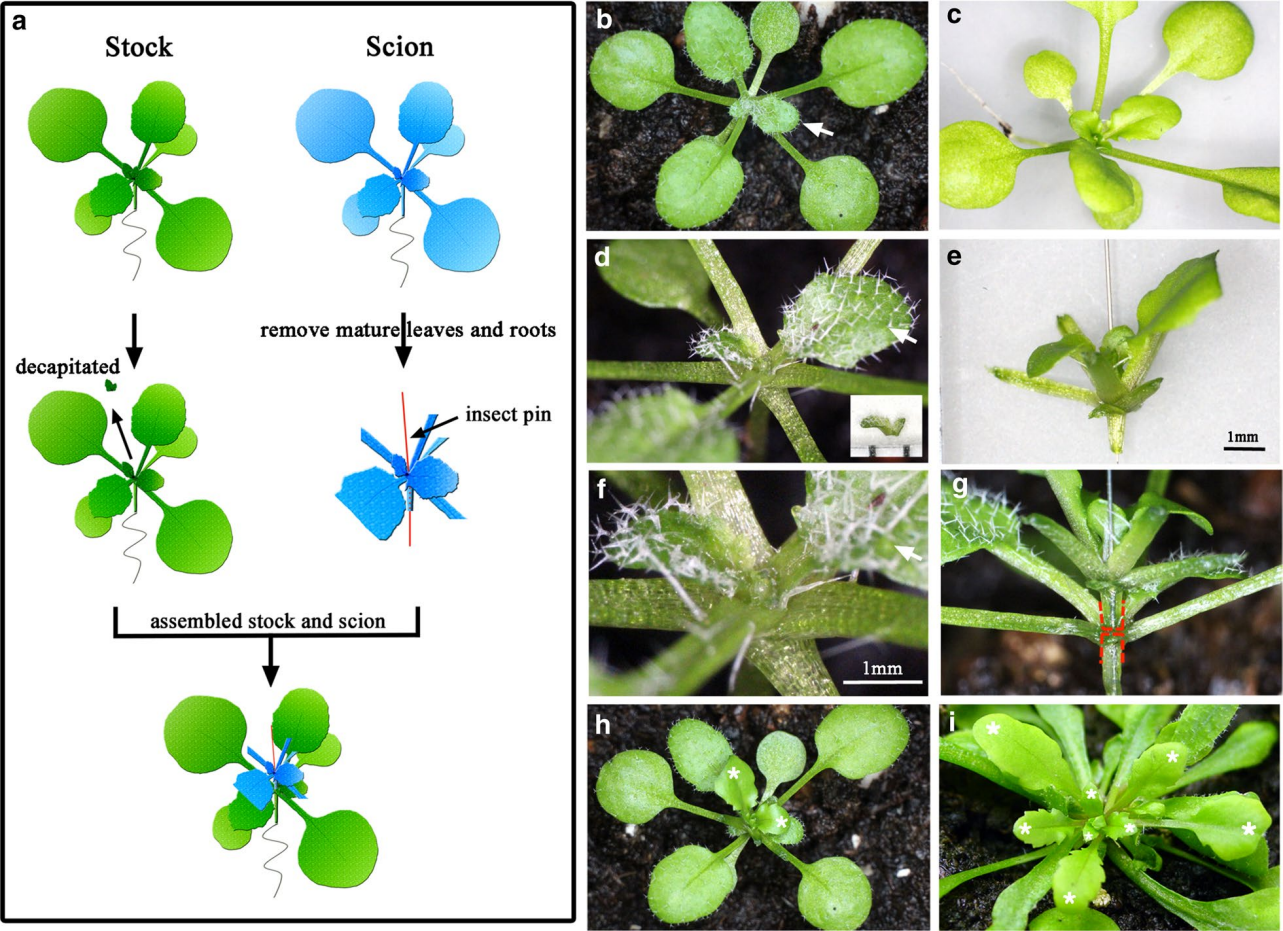
Arabidopsis pin-fasten grafting under long-day (LD) growth conditions. **a** Arabidopsis pin-fasten grafting. Ten- to 12-day-old Arabidopsis seedlings were decapitated from apical meristems (stocks *green*) and pin-fastened with scions (*blue*). The insect pin is indicated by a *red line*. **b**, **c** Images of 10- to 12-day-old LD-grown Arabidopsis seedlings used as stocks (**b**) or scions (**c**). **d** Apex-removed stock. The removed apex is shown in the *inset*. *Scale* is 1 mm. Note that only the apex and emerged young leaf primodia were removed. **e** Scions with an insect pin inserted from the base of the petiole through the hypocotyl. **f** Magnified images of apex-removed stocks to show the diameter of a cut area. *Scale bar* is 1 mm. **g**, **h** Grafted plants with a *gl1*-*1* scion assembled on a wild-type stock. **i** Image of successful grafting plants at 14 days after grafting. The 6th leaf of the stocks is indicated by a *white arrow* in (**b**, **d**, **f**). *Red dashed lines* in (**g**) represent the stems of scions (*upper*) and stocks (*lower*). *White asterisks* in (**h**, **i**) indicate the original and newly developed leaves of *gl1*-*1* scions.

We first performed Arabidopsis pin-fasten grafting under LD conditions with soil-grown 10- to 12-day-old seedlings. The scions were grafted onto epicotyls of apex-removed stocks. The mature leaves of the stocks remained intact, which provided sufficient source strength to transport nutrients and signaling molecules to scions (Figure 1a). The plants at this stage usually contain 4 well-developed leaves and begin developing the 5th and 6th leaves (Figure 1b, c). To provide the phenotypic markers for discriminating scions from stocks after grafting, we used Arabidopsis *glabrous1*-*1* mutants (*gl1*-*1*, a trichrome-less mutant) as scions to graft onto wild-type (Columbia ecotype) or P*35S*-*GUS* transgenic stocks [8]. Previous results showed that *GL1* and *GUS* act cell-autonomously and so are useful markers to distinguish scions from stocks after grafting [8, 28].

The grafting was conducted with a flat-surface method. The apices of stocks were horizontally cut above the 6th leaf to remove the apical meristem and keep mature and developing leaves intact (Figure 1b, d, f). We usually cut out an apex with a leaf primodia, which is 0.5 mm in diameter (Figure 1d, inset), to match the diameter of the scion hypocotyls for full contact (Figure 1e). The scions were horizontally cut from hypocotyls, and leaves >0.3 cm (usually the first and second pair of leaves) were removed (Figure 1c, e). To assemble the stocks and scions, an insect pin was inserted into a scion from the base of petiole (to avoid damaging the apical meristem of scions) through the hypocotyl (Figure 1e). The inserted scions were tightly attached to the flat surface of stocks (Figure 1g, h). The assembled plants were kept in a sealed chamber to maintain humidity for 1 week, then transferred to a growth chamber for further manipulation. To avoid a great change in humidity, the lid on the sealed chamber was removed gradually.

In some cases, emerging axillary buds from stocks may push away the scions and result in failure to develop a vascular connection between scions and stocks. To reduce the development of axillary buds from stocks, the diameters of the cut surface on stocks and scions were as similar as possible to ensure complete contact between scions and stocks after grafting (Figure 1g).

At 14 days after grafting, successfully grafted plants were easily recognized, with newly differentiated glabrous leaves observed on grafted plants (Figure 1i). Most of the scions that failed to differentiate were withered within a few days after grafting, with no glabrous leaves observed. Typically, an experienced researcher can easily perform at least 15 grafts per hour. Among the 464 grafts we have conducted, the success rate of grafting was 66% (305/464), so this technique is a simple and robust grafting method.

### Arabidopsis pin-fasten grafting under short-day (SD) growth conditions

**Figure 2:**
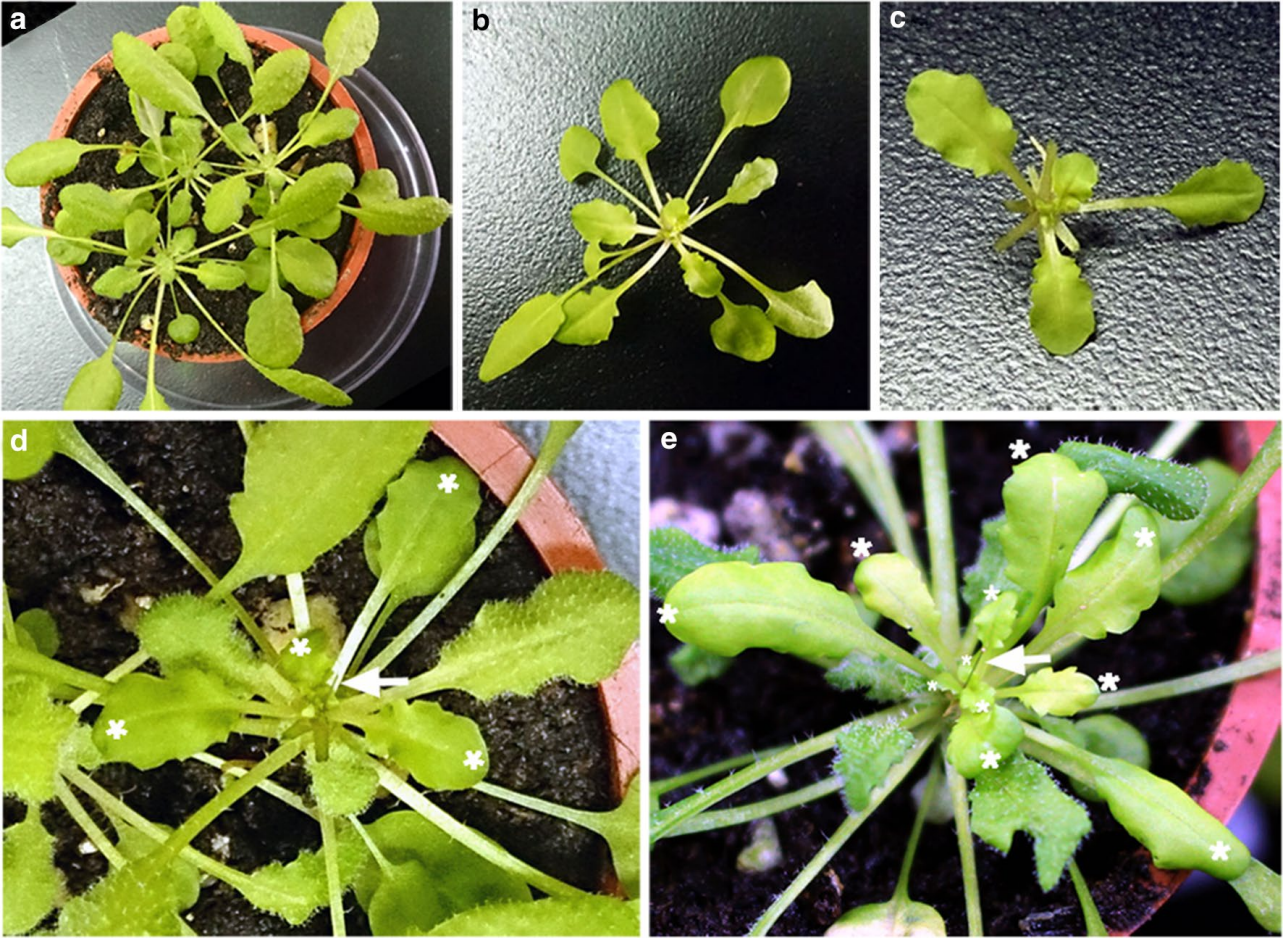
Arabidopsis pin-fasten grafting under short-day (SD) growth conditions. Forty-five-day-old SD-grown Arabidopsis wild-type (Col) and *gl1*-*1* plants were used as stocks (**a**) and scions (**b**), respectively. **c** *gl1*-*1* scions with mature leaves removed. **d** *gl1*-*1* scions were pin-fastened to Col stocks. The glabrous leaves of scions are indicated by *white asterisks*. **e** Successfully grafted plant at 2 weeks after grafting. The newly developed glabrous leaves are indicated by *white asterisks*. The insect pins are indicated by *white arrows.*

LD-grown Arabidopsis develops rapidly, which restricts the use of grafting to examine systemic signals in meristem differentiation. To broaden the application of pin-fasten grafting, we used SD-grown Arabidopsis for grafting experiments. Under this condition, Arabidopsis plants grow slowly, which provides sufficient time to analyze systemic signals. At 15 days under SD conditions, the seedlings of wild-type or *gl1*-*1* Arabidopsis produced only 1–2 leaves and slender hypocotyls, which were difficult to use for pin-fasten grafting. At 30 or 45 days, the plants usually developed 5–6 or 12–15 leaves, respectively. The leaf number of 30-day-old SD-grown plants was equivalent to that of 2-week-old LD-grown plants, which suggests a similar developmental stage. Of 95 grafts we conducted, the success rate of grafting was 73% (69/95). Therefore, the pin-fasten grafting is applicable for SD-grown seedlings. To examine the grafting capacity at later developmental stages, we used 45-day-old SD-grown plants for pin-fasten grafting (Figure 2a). Similar to LD-grown grafting, the pin-inserted scions were fastened on top of apex-removed stocks (Figure 2b–d). The assembled grafted plants were kept in the sealed chamber for 1 week to maintain humidity, then transferred to a growth chamber. At 2–3 weeks after grafting, newly developed glabrous leaves were observed in scions (Figure 2e). Among 65 grafts, the success rate was 50% (32/65), which suggests that older SD-grown Arabidopsis still have high capacity for regenerating graft junctions. Thus, stocks and scions at broad developmental stages are suitable for pin-fasten grafting.

### Use of Arabidopsis pin-fasten grafting for hypocotyl grafting

**Figure 3:**
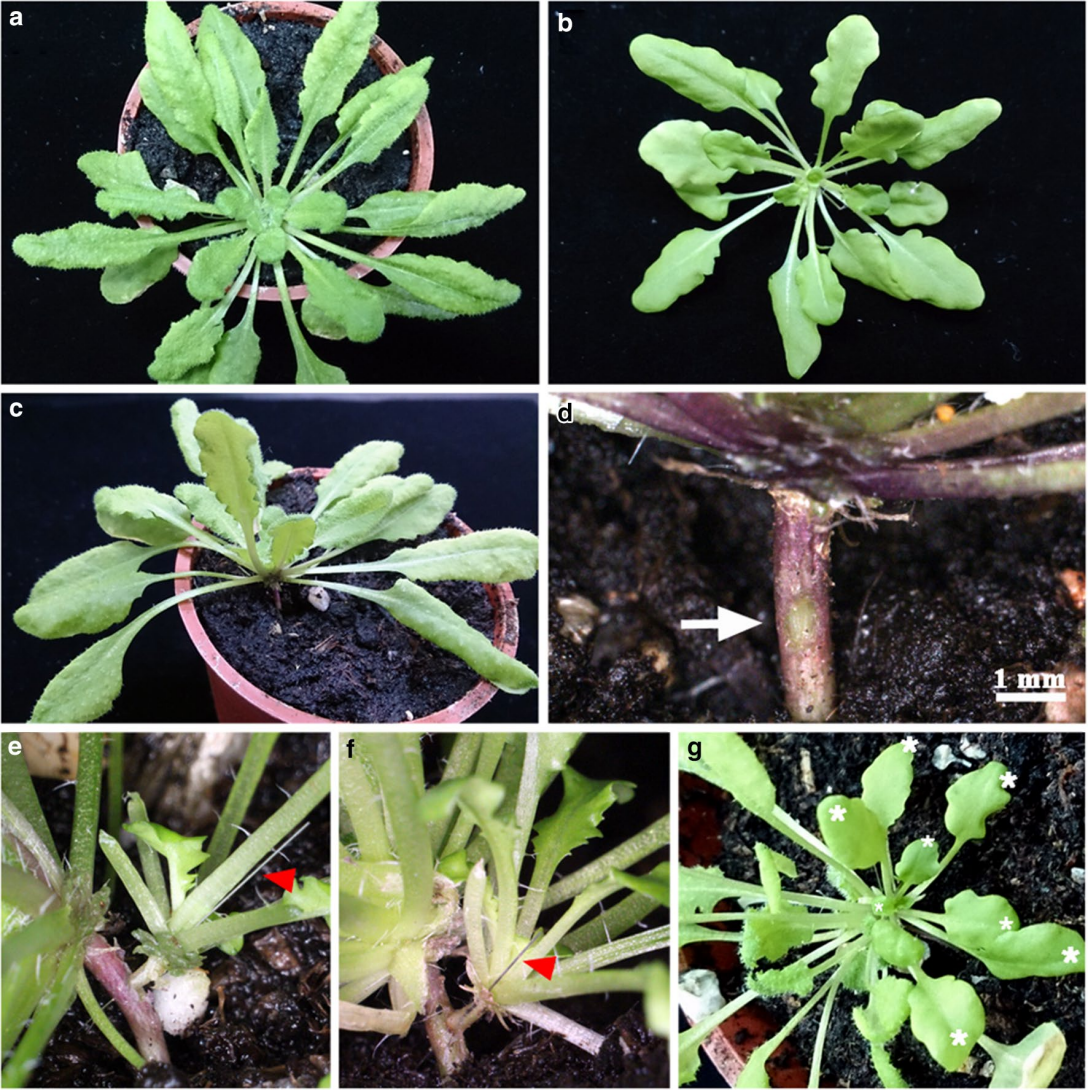
Use of Arabidopsis pin-fasten grafting for hypocotyl grafting. Two-month-old SD-grown Arabidopsis wild-type (Col) and *gl1*-*1* plants were used as stocks (**a**) and scions (**b**), respectively. **c** Two to 3 leaves near the hypocotyl of stock were removed to expose the hypocotyl. **d** The epidermis of the hypocotyl was removed by use of blades. The scraped epidermis is indicated by an *arrow*. *Scale bar* is 1 mm. **e** *gl1*-*1* scions (*right*) were pin-fastened on stock hypocotyls (*left*). The insect pins are indicated by *red arrowheads*. **f**, **g** Images of successful grafts at 2 weeks after grafting. The newly developed glabrous leaves of scions are indicated by *white asterisks* in (**g**).

Arabidopsis hypocotyl grafting was previously conducted with young seedlings [8]. To further extend the application of our method, we examined whether mature plants could be pin-fasten grafted onto hypocotyls. We used 2-month-old SD-grown Arabidopsis for hypocotyl grafting (Figure 3a, b). We removed 2–3 mature leaves near the grafting site on stock hypocotyls to expose hypocotyls (Figure 3c). A blade was used to scrape stock hypocotyls to remove the epidermis (Figure 3d). An insect pin was inserted into scions and attached firmly onto the surface of stock hypocotyls (Figure 3e). At 14 days after grafting, newly developed glabrous leaves were observed in scions (Figure 3f, g). Among 32 grafts, 29 successfully developed new leaves (success rate was 91%), so pin-fasten grafting can be used for hypocotyl grafting. We also found that the pin-fasten grafting could be used with 10- to 12-day-old LD-grown Arabidopsis seedlings (Additional file 1: Figure S1). In our 44 grafts, the success rate was 48% (21/44).

The summary of pin-fasten grafting experiments conducted at different conditions is in Table 1.

**Table 1:** Summary of Arabidopsis pin-fasten grafting experiments

	Age (days)	Leaf number	Succeed/total grafts	Success rate (%)
Epicotyl grafting				
LD	10–12	5–6	305/464	66
SD	30	5–6	69/95	73
SD	45	12–15	32/65	50
Hypocotyl grafting				
LD	10–12	5–6	21/44	48
SD	63	21–28	29/32	91

### Development of vascular connection between stocks and scions with pin-fasten grafting

**Figure 4:**
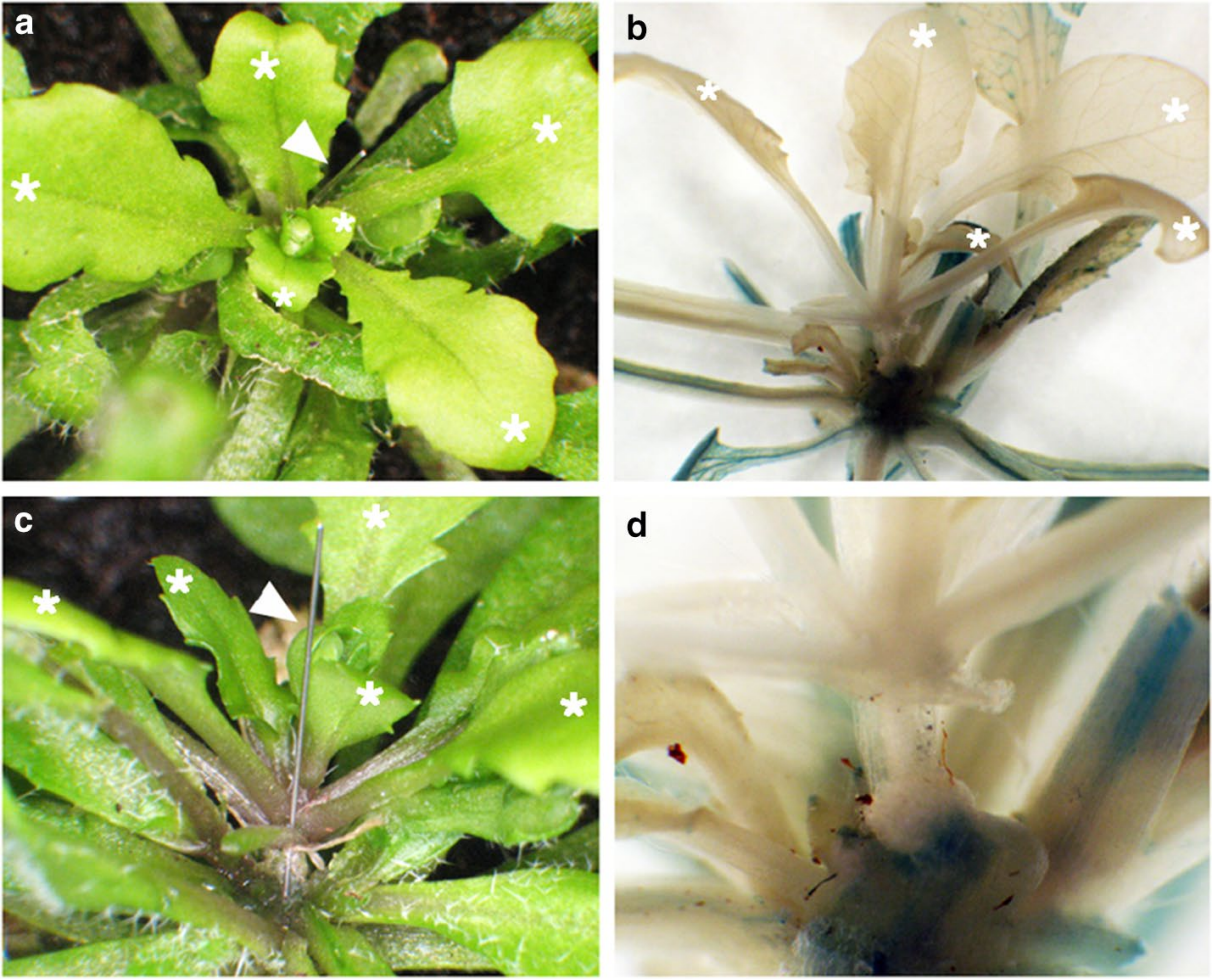
Differentiation of *gl1*-*1* scions in successfully grafted *P35S*-*GUS* transformant stocks. **a**, **c** Arabidopsis pin-fasten grafted plants at 14 days after grafting under LD conditions. Arabidopsis *gl1*-*1* scions were grafted onto *P35S*-*GUS* transformant stocks. Trichrome-less leaves were developed from *gl1*-*1* scions (*white asterisks*) and trichrome-containing leaves were from *P35S*-*GUS* transformant stocks. Insect pins are indicated by *arrowheads*. **b**, **d** Histochemical staining of Arabidopsis *gl1*-*1* scions grafted onto *P35S*-*GUS* transformant stocks at 14 days after grafting. GUS activity was detected in stocks but not scions.

We further investigated the differentiation of scions and stocks on the grafted junction by histochemical staining (Figure 4). At 14 days after grafting, which *gl1*-*1* scions were grafted onto P35S-*GUS* stocks under LD conditions, the newly differentiated glabrous leaves were observed from *gl1*-*1* scions (Figure 4a, c). Histochemical assay showed GUS activity exclusively detected in P35S-*GUS* stocks but not glabrous scions (Figure 4b, d), which is consistent with GUS acting cell-autonomously [28].

**Figure 5:**
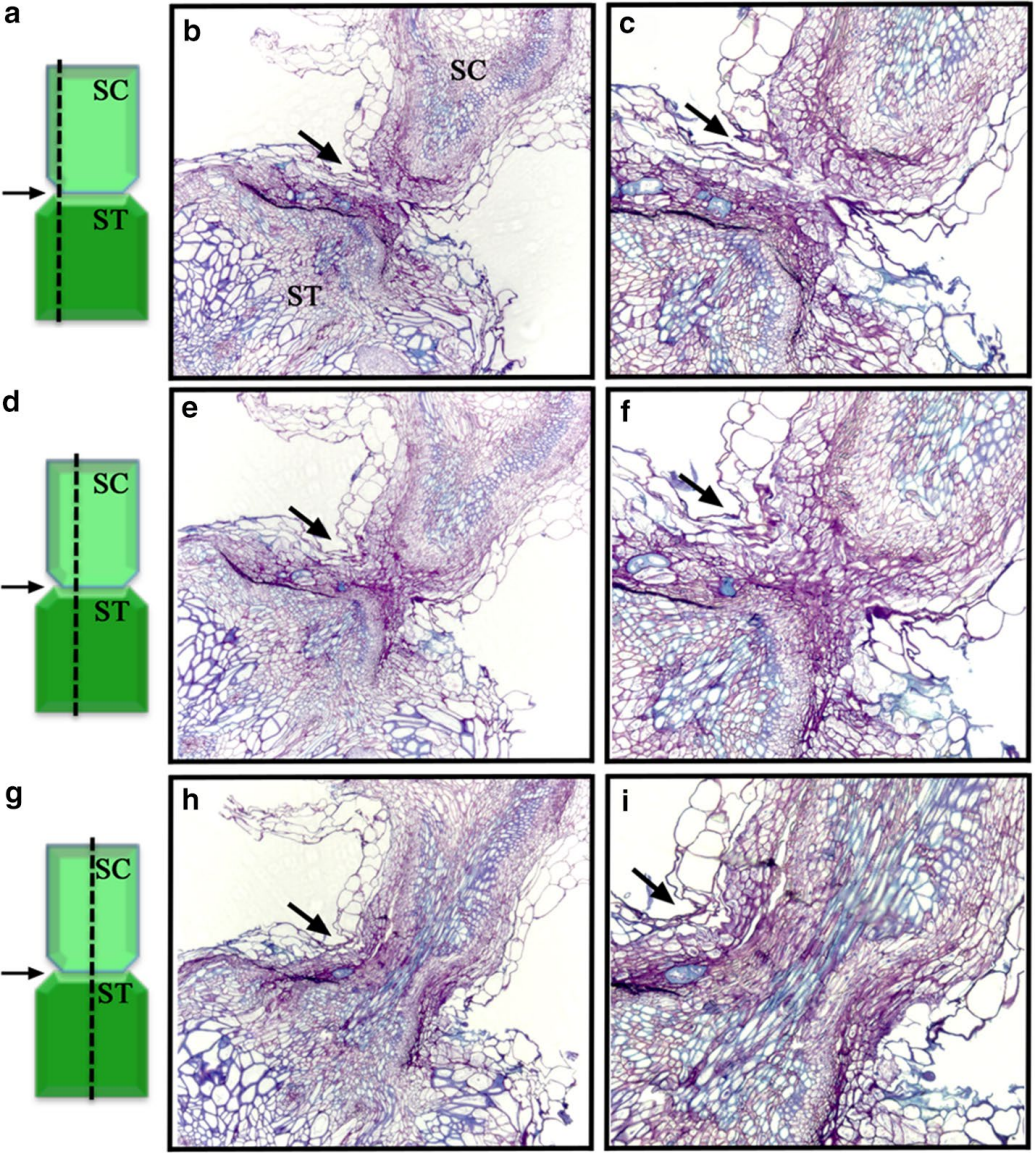
Longitudinal sections showing connected vascular tissues between scions and stocks. Toluidine-blue staining of longitudinal sections. Cells with primary cell wall are stained *pink*, and cells with secondary cell wall (vascular tissues) are stained *blue*. Cells stained *blue* were well connected across the graft junction. The graft junction is indicated by *arrows*. **a**, **d**, **g** Longitudinal sections of Arabidopsis *gl1*-*1* scions (SC) grafted onto *P35S*-*GUS* transformant stocks (ST). The position of the longitudinal section, near epidermal cells (**a**–**c**), inner cell layers (**d**–**f**), or vascular tissues (**g**–**i**), is indicated by a *dashed line* on the *left.*

To further confirm the establishment of a vascular connection between stocks and scions, we obtained longitudinal sections to visualize the vasculature across the grafted junctions. The graft junction between stocks and scions was cut from the successfully grafted plants at 14 days after grafting. The leaves surrounding graft junctions were removed to reveal the grafted tissues. Tissues were embedded in LR5 resin. After sectioning, tissues were stained with Toluidine blue, which differentially stains primary cell walls pink and secondary cell walls of xylem blue. Continuous longitudinal sections from tissues near epidermal cells revealed regenerated cells that were connected at the graft junction (Figure 5a–f). Sections close to vascular tissues showed continuous blue cells throughout the graft junction, which indicated that the vascular tissues were well connected between stocks and scions (Figure 5g–i).

### Development of symplastic connection between stocks and scions

To examine phloem translocation between scions and stocks, we used a fluorescent phloem-limited dye, 5(6)-carboxyfluorescein diacetate (CFDA), to monitor the development of a functional symplastic connection. CFDA has been extensively used as a marker to visualize phloem transport [29]. After CFDA is introduced into plant cells, the acetate groups are removed by intracellular esterases to convert into carboxyfluorescein (CF). The negative-charged CF no longer transports through the plasma membrane but is retained within cells. However, because of the relatively small size of CF (376 Da), it allows CF to freely diffuse through plasmodesmata into phloem and follow the phloem translocation stream to spread all over plants [30]. Thus, the detection of fluorescent signals in systemic leaves indicates a functional symplastic connection.

**Figure 6:**
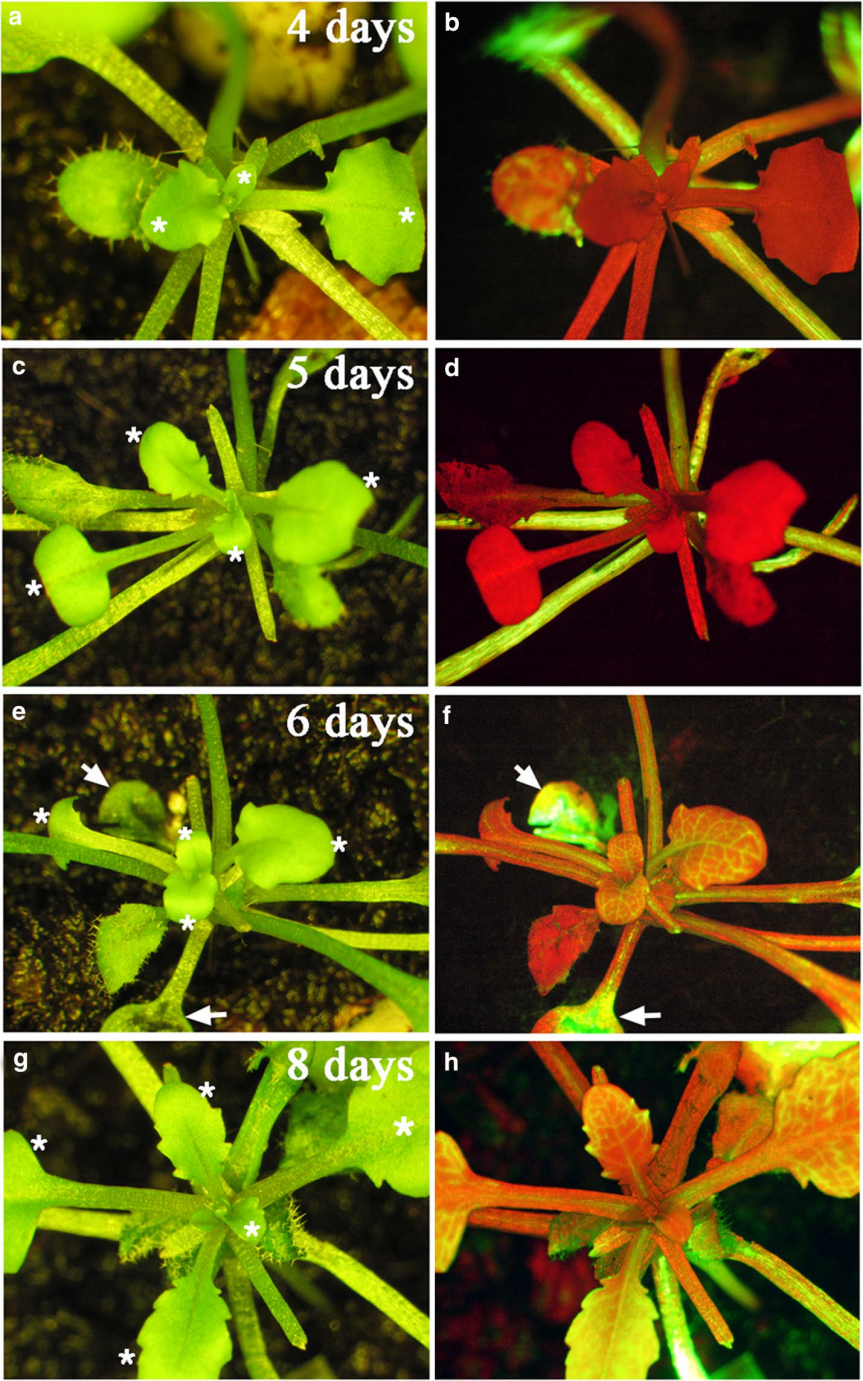
Development of symplastic connection between stocks and scions. Arabidopsis *gl1*-*1* scions were grafted onto wild-type stocks under LD conditions. The 5(6)-carboxyfluorescein diacetate dye (*green fluorescence*) was introduced onto leaves (indicated by *arrows*) of wild-type stocks at different times after grafting. The images (*left panels*) and florescence images (*right panels*) were taken at 4 days (**a**, **b**), 5 days (**c**, **d**), 6 days (**e**, **f**), and 8 days (**g**, **h**) after grafting. Note *green fluorescent* signals (or *yellow colors* when merged with *red* *florescence*) in the vasculature of *gl1*-*1* scions at 6 or 8 days after grafting but not 4 or 5 days after grafting. The *red colors* in *florescence* images represents the auto-florescence of chlorophyll. The glabrous leaves of scions are indicated by *white asterisks* (*left panels*).

Previously, CFDA has been used to examine phloem translocation between stocks and scions in Arabidopsis micrografting [31, 32]. To examine the establishment of functional phloem connection in pin-fasten grafting, we introduced CFDA into the mature leaf of LD-grown stocks at different times after grafting. Because most of the grafts that failed to differentiate were withered at early stages after grafting, we selected vigorous scions for CFDA experiments to ensure these scions were potentially successful grafts. At 3–5 days after grafting, fluorescence microscopy revealed highly accumulated fluorescence signals in stocks, including petioles, hypocotyls, and leaves, but not in *gl1*-*1* scions (Table 2; Figure 6a–d). However, at 6–8 days after grafting, fluorescence signals were observed both in wild-type stocks and vasculature of *gl1*-*1* scions (Table 2; Figure 6e–h). Among the grafted plants examined, the detection of fluorescent signals from scions was 0% at 3–5 days after grafting but 17% at 6 days after grafting (Table 2). As scions grew, the detection rate of fluorescence signals in scions increased to 76% at 7 days after grafting and 89% at 8 days (Table 2). Therefore, the symplastic connection between LD-grown scions and stocks began to be established at 6 days after grafting, with the symplastic connection was established at 8 days after grafting in most grafted plants. These results are consistent with previous finding that the functional phloem connection is established at 7- to 10 days after grafting [31]. However, in recent analyses of vascular reconnection in Arabidopsis micrografting, when CFDA was introduced to cotyledons of scions, fluorescence was detected in the roots of stocks at 3 days after grafting [32]. As the grafting methods are different in these experiments (Y-shape micrografting [31], butt alignment micrografting [32]), it is possible that the timing of vascular reconnection is various in different grafting methods or the position of grafted tissues may affect the phloem translocation. Further experiments are required to examine these possibilities.

**Table 2:** Number of plants with symplastic connection between stocks and scions

Days after grafting	3	4	5	6	7	8
Total no. of plants	14	16	20	24	37	18
CFDA+	0	0	0	4	28	16
CFDA−	14	16	20	20	9	2
Rate of detection (%)	0	0	0	17	76	89

*CFDA* 5(6)-carboxyfluorescein diacetate.

**Figure 7:**
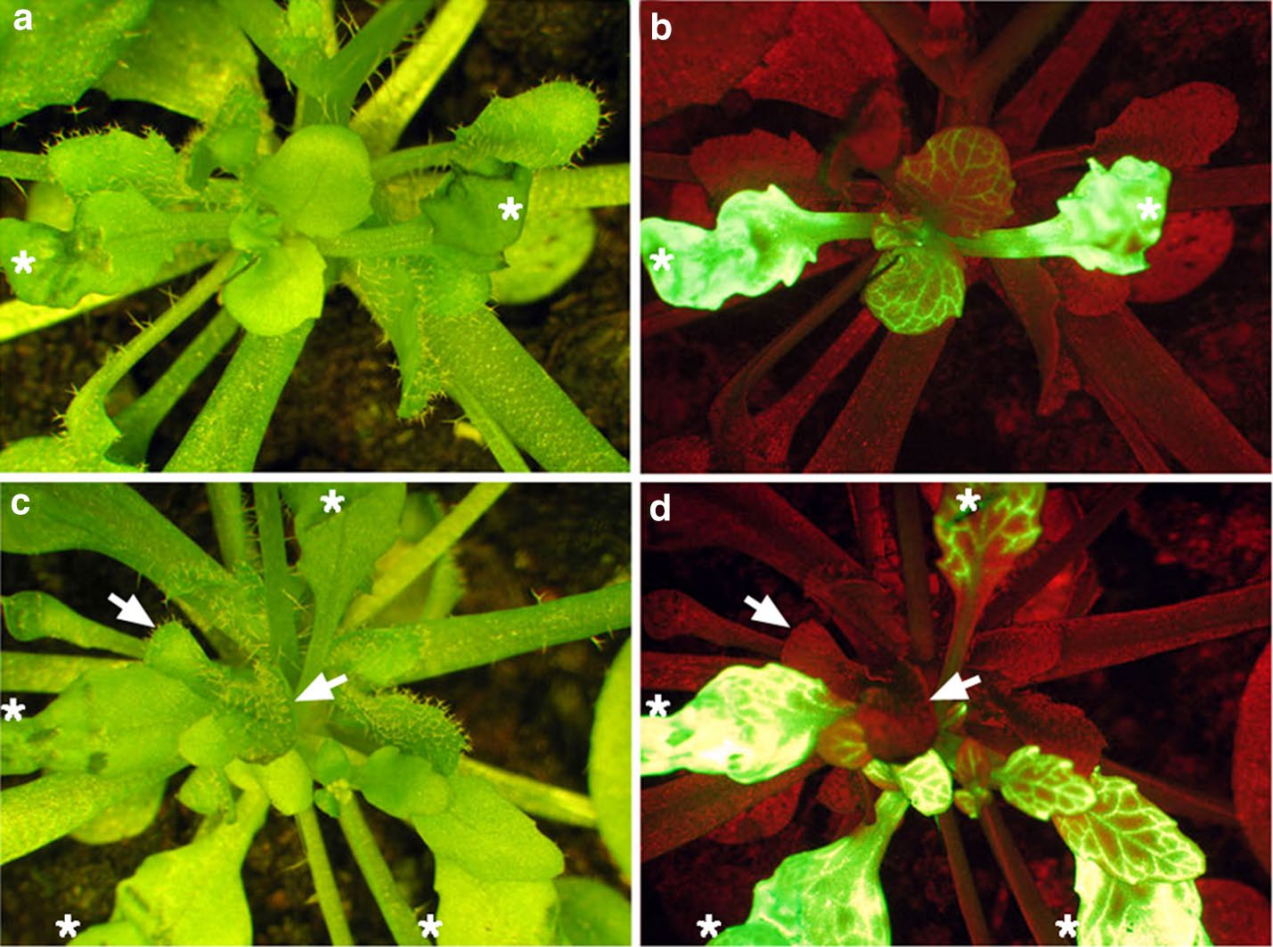
The direction of phloem translocation between stocks and scions. Arabidopsis *gl1*-*1* scions were grafted onto wild-type stocks under LD conditions. The 5(6)-carboxyfluorescein diacetate dye (*green fluorescence*) was introduced onto leaves of *gl1*-*1* scions (*white asterisks*). The images (**a**, **c**) and florescence images (**b**, **d**) were taken at 1 h after dye injection. The sink tissues of stocks (leaf primodia and apex) are indicated by *arrows*. Note that green fluorescent signals were not detected in these tissues. The *red colors* in florescence images represents the auto-florescence of chlorophyll.

To examine whether phloem translocation also occurs from scions to stocks in epicotyl grafting, we introduced CFDA into the leaves of LD-grown scions at 10 days after grafting (Figure 7). At this stage, the symplastic connection between stocks and scions is well established (Figure 6g, h). At 1 h after dye injection, fluorescent signals were observed in injected leaves and young leaves of scions but not mature leaves or young leaves of stocks (Figure 7). Of 28 scions introduced, no fluorescent signals were observed in stocks. Thus, the phloem translocation may be from the dye-injected scion leaves to the scion apex but not to sink tissues of stocks. Indeed, these results are consistent with the phloem translocation being affected by phyllotaxy and proximity of the source to the sink tissues [24–26]. However, as the leaves of scions at this stage may not be fully differentiated, it is possibility that the phloem translocation may occur from scions to stocks at later stages.

Our system provides a useful tool to analyze leaf-derived mobile signals in Arabidopsis. In our approach, the scions and stocks used for grafting were soil-grown. Sterile conditions are not required for the grafting, which reduces the humidity shock on transferring grafted plants from sterile conditions to soil.

### Protocol

The Arabidopsis seedling grafting was performed under a dissecting stereomicroscope (OPTIMA ZM-160AT).

1. Arabidopsis seeds were grown on soil under long-day (LD) conditions for 10–12 days or under short-day (SD) conditions for 30 days until the 5th and 6th leaves began to develop. The apices of stocks, which usually contain the 7th or 8th leaf primodia, were removed by use of microdissecting spring scissors (Fine Science Tools, catalog no. 15001-08). The diameter of removed stock apices was as similar as possible to that of scion hypocotyls, which are usually about 0.05 cm at this stage (Figure 1d, e).
2. Seedlings that served as scions were grown under the same conditions as stocks. The mature leaves of scions were removed, and hypocotyls were cut from 0.1 cm below cotyledons with use of a two-edge razor blade to remove roots and most of the hypocotyls. A 0.1-mm diameter insect pin (Fine Science Tools, catalog no. 26002-10) was inserted from the base of the leaf-removed petiole (to avoid damaging the apical meristem of scions) through the hypocotyl of scions (Figure 1e).
3. The stocks and scions were assembled by inserting the insect pin (Figure 1g). The surface of excised tissues should contact tightly. The grafted plants were transferred to a tray with a lid to retain humidity. The trays were returned to the same growth conditions for 7 days. However, the plants should not be overflowing to prevent the formation of adventitious roots on scions.
4. Seven days after grafting, the lid of tray was gradually removed to minimize humidity shock. Success grafts were observed with new leaves produced on scions.

## Materials

### Plant materials and growth conditions

*Arabidopsis thaliana* Columbia-0 (Col), P*35S*-*GUS* transgenic plants (in Col background) and *glabrous1*-*1* (*gl1*-*1*) mutant plants were used for grafting. Col and *gl1*-*1* seeds were obtained from the Arabidopsis Biological Resource Center (ABRC, Columbus, OH, USA). Plants were grown under LD (16/8 h) or SD conditions (8/16 h), with 22°C/20°C day/night cycles and light intensity 100 μmol m^−2^ s^−1^.

### Histological analysis

To distinguish scions from stocks, *gl1*-*1* plants were used as scions and grafted onto *P35S*-*GUS* transformant stocks. Two weeks after grafting, successfully grafted plants were harvested and incubated in GUS staining solution (50 mM sodium phosphate, pH 7.0, 10 mM EDTA, 0.5 mM potassium ferricyanide, 0.5 mM potassium ferrocyanide, 1 mM X-Gluc, 0.01% Triton X-100) at 37°C for 16 h. Stained tissues were incubated in 95% ethanol to remove chlorophyll, then photographed under a Leica Z16 Apo microscope. To examine the connection of vascular tissues between stocks and scions, graft junctions were cut and embedded in LR5 resin. Continuous longitudinal sections 2 μm thick were obtained and stained with 1% Toluidine blue.

### 5(6)-Carboxyfluorescein diacetate (CFDA) labeling

Phloem limited dye, CFDA (Molecular Probes), was applied as described [29] with the following modifications. CFDA was dissolved in dimethyl sulfoxide (DMSO) as 13 mM stock, which was diluted to 0.13 mM before being applied to leaves. The leaves of stocks were crimped by use of forceps and a 5-μl drop of CFDA was loaded on the adaxial surface of leaves. After 1 h, plants were examined under a fluorescent dissecting microscope (Leica Z16 APO) with excitation and emission wavelength 492 and 517 nm, respectively, to visualize fluorescence signals.

## Additional file

Additional file 1:
**Figure S1.** Use of pin-fasten grafting for Arabidopsis hypocotyl grafting. Twelve-day-old LD-grown Arabidopsis wild-type (Col) and *gl1*-*1* plants were used as stocks and scions, respectively. (A) *gl1*-*1* scions (right) were pin-fastened on wild-type stock hypocotyls (left). (B) Images of successful grafts at 2 weeks after grafting. The insect pins are indicated by white arrows. The glabrous leaves of scions are indicated by white asterisks.

## Abbreviations

CFDA: 6(5)-carboxyfluorescein diacetate
CF: carboxyfluorescein
DMSO: dimethyl sulfoxide
