# Supplementary figures and images for: A pin-fasten grafting method provides a non-sterile and highly efficient method for grafting Arabidopsis at diverse developmental stages
Source: Plant Methods. 2015 Jul 8;11:38. doi: 10.1186/s13007-015-0081-7 (PMC4495618; doi:10.1186/s13007-015-0081-7)

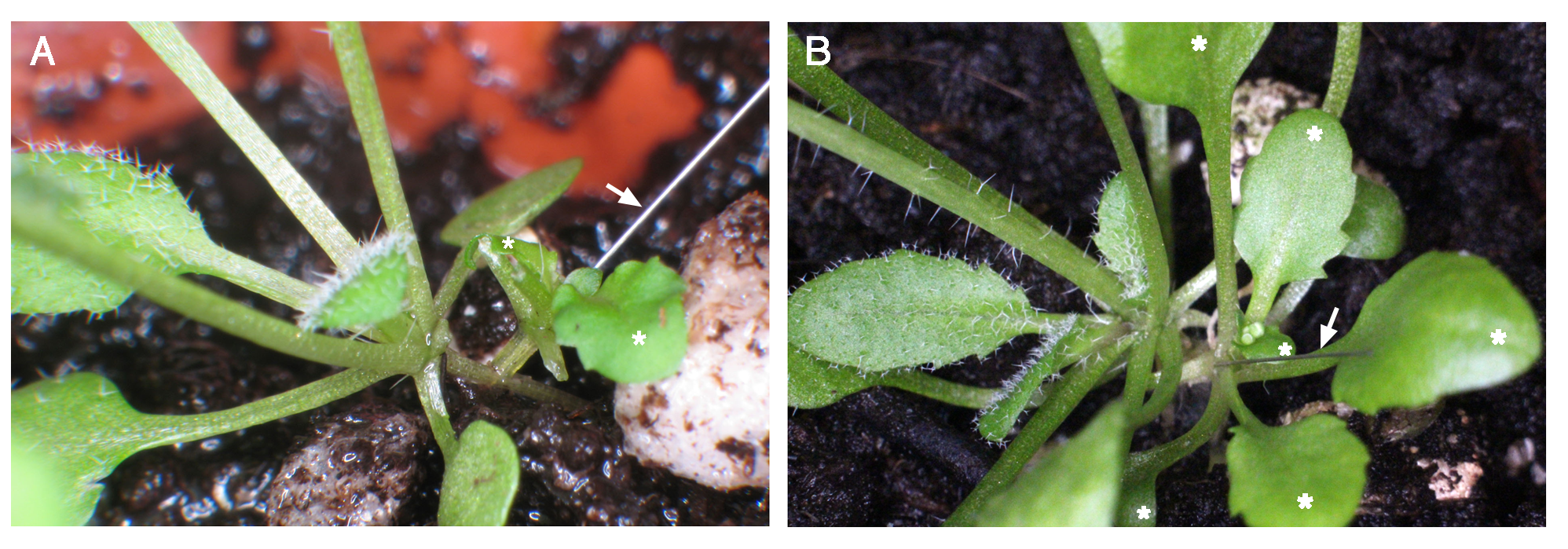

Supplement: Additional file 1: — Figure S1. Use of pin-fasten grafting for Arabidopsis hypocotyl grafting. Twelve-day-old LD-grown Arabidopsis wild-type (Col) and gl1-1 plants were used as stocks and scions, respectively. (A) gl1-1 scions (right) were pin-fastened on wild-type stock hypocotyls (left). (B) Images of successful grafts at 2 weeks after grafting. The insect pins are indicated by white arrows. The glabrous leaves of scions are indicated by white asterisks. [file 13007_2015_81_MOESM1_ESM.tif]
